# Growth-Inhibitory Effect of Chicken Egg Yolk Polyclonal Antibodies (IgY) on Zoonotic Pathogens *Campylobacter jejuni*, *Salmonella* spp. and *Escherichia coli*, In Vitro

**DOI:** 10.3390/ijms26031040

**Published:** 2025-01-25

**Authors:** Paulina Czoska, Karolina Tarsalewska, Magdalena Ponichtera, Magda Rybicka, Natalia Sowa-Rogozinska, Hanna Sominka-Pierzchlewicz, Aleksandra Stodolna, Patrycja Ogonowska, Aleksandra Kosciuk, Renata Glosnicka, Krzysztof Piotr Bielawski

**Affiliations:** 1 Research and Development Department of Salmonella Center IMMUNOLAB Ltd., Kladki 24, 80-822 Gdansk, Poland; p.czoska@immunolab.com.pl (P.C.); k.tarsalewska@immunolab.com.pl (K.T.); m.ponichtera@immunolab.com.pl (M.P.); n.sowa-rogozinska@immunolab.com.pl (N.S.-R.); h.sominka@immunolab.com.pl (H.S.-P.); a.stodolna@immunolab.com.pl (A.S.); p.ogonowska@immunolab.com.pl (P.O.); a.kosciuk@immunolab.com.pl (A.K.); r.glosnicka@immunolab.com.pl (R.G.); 2 Intercollegiate Faculty of Biotechnology, University of Gdansk and Medical University of Gdansk, Abrahama 58, 80-307 Gdansk, Poland

**Keywords:** campylobacteriosis, *Campylobacter jejuni*, *Salmonella* spp., *Escherichia coli*, zoonotic pathogens, immunoglobulin Y, IgY, passive immunization, growth inhibition, antibiotic alternatives

## Abstract

The overuse of antibiotics in animal husbandry has driven the search for alternative strategies to combat zoonotic pathogens. Foodborne zoonotic diseases caused by pathogenic bacteria pose a significant threat to human health, and therefore food safety should be a priority. This study investigates the in vitro inhibitory effects of chicken egg yolk immunoglobulin Y (IgY) on the growth and viability of three major foodborne pathogens: *Campylobacter jejuni*, *Salmonella* spp., and *Escherichia coli*. IgY was isolated from immunized hen egg yolks using a modified water dilution method, and its antigen-specificity confirmed through agglutination assays. Growth inhibition was evaluated across multiple doses and time points, revealing a dose-dependent bacteriostatic effect against all tested pathogens. A single dose of IgY (0.5 mg/mL) significantly reduced *C. jejuni* counts by up to 7 log, while repeated doses were required for *Salmonella* spp. and *E. coli*. These findings highlight egg yolk immunoglobulin’s potential as a source of sustainable, effective, ethical, readily available, and inexpensive antibiotic substitutes in livestock management. Future research will focus on validating these results in vivo and exploring large-scale production of IgY for practical application in animal healthcare.

## 1. Introduction

The One Health approach proposed by the World Organization for Animal Health (WOAH) is based on the fact that human health is highly dependent on animal health [1]. Approximately 60% of human pathogens are zoonotic, transmitted through domestic or wild animals. These zoonotic pathogens should be targeted and controlled at animal population level to prevent human infections. Foodborne zoonotic infections caused by pathogenic bacteria such as *Campylobacter jejuni*, *Salmonella* spp., and Shiga toxin-producing *Escherichia coli* (STEC) represent significant threats to public health. High-risk groups include the elderly, children, women of childbearing age, and immunocompromised individuals [2]. The One Health concept approach identifies the need for intensive research in three main areas: Foodborne Zoonoses (FBZ), Antimicrobial Resistance (AMR), and Emerging Threats (ET). According to the European Food Safety Authority (EFSA) and the European Centre for Disease Prevention and Control (ECDC), campylobacteriosis was the most frequently reported zoonotic infection in 2020 (60%, 120,946 reported incidences). The second most frequent was salmonellosis (26.1%, 52,706 cases), followed by yersiniosis (2.8%) and STEC infections (2.2%, 4446 cases) [3]. As a consequence of the COVID-19 pandemic, a decrease in the number of zoonoses was recorded in 2020, due to the limited availability of healthcare and requests for treatment of zoonoses, and restriction of treatment to the most severe cases. The lower incidence was also the result of reduced travel and events, restaurant closures, social distancing, better hygiene, and hand-washing habits.

The overuse of antibiotics in livestock is contributing to the emergence of drug-resistant bacteria, a dangerous and growing global health threat [4]. There is a high risk that the incidence of zoonotic diseases will increase due to tighter restrictions on the use of antibiotics in livestock and the lifting of COVID-19 restrictions. Developing and implementing effective alternatives to antibiotics in animal healthcare has become a pressing necessity [5,6]. One such approach includes the use of immunoglobulin Y (IgY), a 180 kDa avian homologue of human 150 kDa immunoglobulin G (IgG). From an animal welfare perspective, the use of laying hens for antibody production is more humane and allows for a reduction in animal use and invasive blood sampling. Moreover, each egg yolk yields approximately 100 mg of IgY, with 2–10% being antigen-specific [7]. Compared to IgG, IgY has distinct advantages: it is more hydrophobic and is resistant to proteolysis, retaining 40% of its activity after 8 h of incubation with chymotrypsin [8]. IgY is stable in the pH range of 4 to 9 and up to 65 °C in aqueous conditions. Chicken IgY antibodies can neutralize toxins, inhibit and destroy bacterial enzymes, and prevent bacterial adhesion to host cells, making them potentially useful in pulmonary and gastrointestinal diseases [9]. Therefore, passive immunization with IgY as a water or feed additive offers an easy-to-implement approach as an alternative to the use of antibiotics in animal husbandry. Moreover, oral administration of IgY to young animals can provide passive protection when their immune systems are still developing and maternal protection is weaning, leaving them vulnerable to infection.

In our research, we demonstrated the in vitro inhibitory effect of IgY, isolated from immunized chicken egg yolks, on zoonotic gastrointestinal pathogens. Specifically, we observed a reduction in the growth and viability of *C. jejuni*, *E. coli*, and *Salmonella* spp. across various IgY dosing regimens.

## 2. Results

### 2.1. Purity Assessment of Isolated IgY by SDS-PAGE (Reducing Conditions)

**Figure 1:**
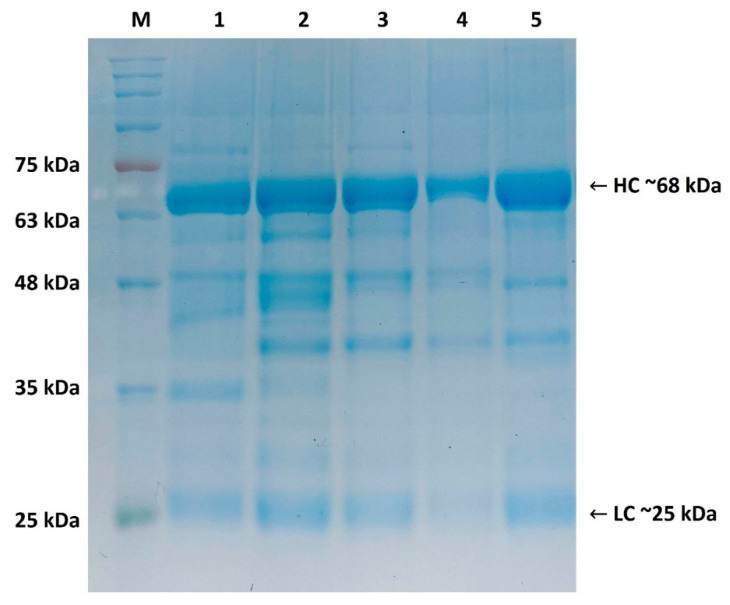
Immunoblotting of IgY antibodies under reducing condition. HC: heavy chains, LC: light chains, M: molecular marker. Lane 1: IgY-CJ, Lane 2: IgY-OB, Lane 3: IgY-OD, Lane 4: IgY-EC, Lane 5: IgY standard; original blot is presented in Supplementary Figure S1.

The method for the isolation and purification of IgY from egg yolk by water dilution, additional delipidation with activated charcoal, and NaCl precipitation followed by filtration proved to be effective, as documented by SDS–PAGE (Figure 1). Four types of isolated IgY were resolved: IgY-CJ (antibodies against *C. jejuni* CJ6 antigen), IgY-OB (antibodies against *Salmonella* O:B antigen), IgY-OD (antibodies against *Salmonella* O:D antigen), and IgY-EC (antibodies against *E. coli* K3 antigen). All resolved IgY types had an expected molecular weight of 68 kDa for heavy chains (HC) and 25 kDa for light chains (LC), as shown in Figure 1. The electrophoretic pattern was consistent with the standard IgY, and was comparable to the type of antigen used for immunization (Section 4.5).

### 2.2. Evaluation of Specific IgY Titers Assessment in Tube Agglutination Test

The titers of specific antibodies against the used antigens were determined by a tube agglutination assay (Section 4.6). IgY titers reached 1:320 for *C. jejuni* CJ6 and *E. coli* K3 antigens, while for *Salmonella* antigens, the titer reached 1:640. The results confirmed the successful production of antigen-specific IgY from immunized hens, and are shown in Table 1.

### 2.3. Growth Inhibition Assay

#### 2.3.1. *Campylobacter jejuni*

**Figure 2:**
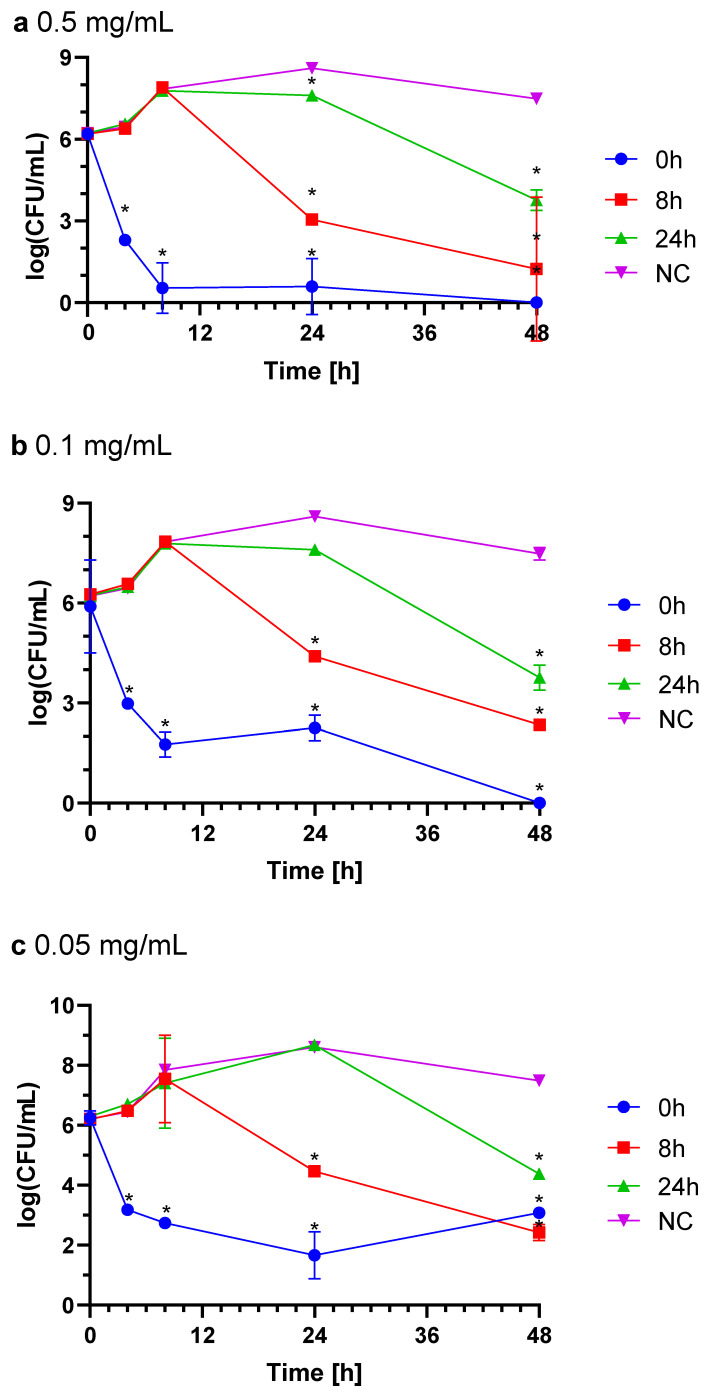
*C. jejuni* growth with different doses of IgY added at time of inoculation (0 h) and 8 h and 24 h after inoculation. IgY final concentration: 0.5 mg/mL (**a**), 0.01 mg/mL (**b**), 0.05 mg/mL (**c**). NC, non-treated control; CFU, colony forming unit. Statistically significant differences compared to the NC are marked with * (*p* < 0.05).

A single dose of IgY at three different concentrations was used in the *C. jejuni* growth inhibition assay, with antibodies added at three time points of incubation (0 h, 8 h, and 24 h). In untreated controls, bacterial counts increased by 2 log CFU over 48 h, whereas treatment with 0.5 mg/mL IgY inhibited the growth of *C. jejuni* CJ6 when added at all three tested time points, resulting in a 6–7 log reduction in bacterial count when IgY was applied at inoculation (0 h) or 8 h, and a 4 log reduction when added at 24 h (Figure 2a). Lower concentrations (0.1 mg/mL and 0.05 mg/mL) showed dose-dependent inhibitory effects, with 6 log and 3–4 log reductions, respectively (Figure 2b,c). The reduction was statistically significant (*p* < 0.05). These findings indicate a strong, dose-dependent bacteriostatic effect of IgY on *C. jejuni*.

#### 2.3.2. *Escherichia coli* (STEC)

**Figure 3:**
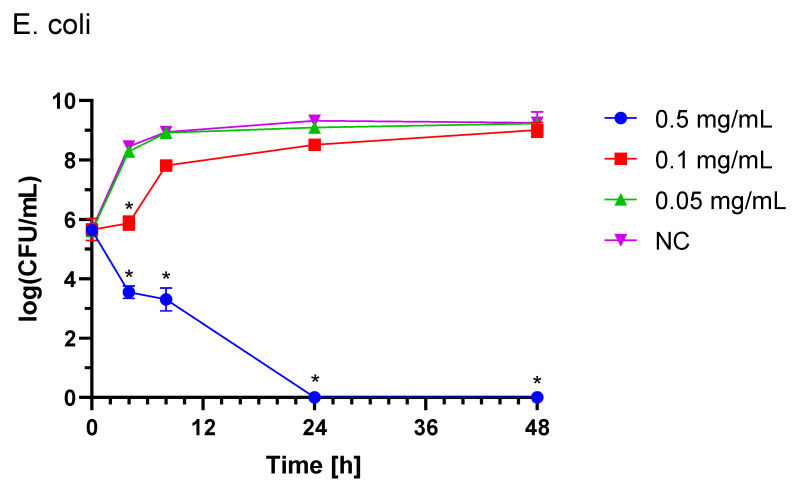
*E. coli* growth with three equal doses of IgY (0.5 mg/mL, 0.1 mg/mL or 0.05 mg/mL) added at time of inoculation (0 h) and 8 h and 24 h after inoculation for *E. coli* K3 (STEC) strain. NC, non-treated control; CFU, colony forming unit. Statistically significant differences compared to the NC are marked with * (*p* < 0.05).

For the *E. coli* STEC strain growth inhibition assay, three doses of three different IgY concentrations were used, with the first dose being added at the time of inoculation, and the second and third dose being added after 8 and 24 h of incubation, respectively. In untreated controls, bacterial counts increased by 3 log CFU, whereas treatment with 0.5 mg/mL resulted in a 6 log CFU reduction (Figure 3). This effect highlights the requirement for higher IgY concentrations and repeated doses to counteract the rapid growth rate of *E. coli*. The reduction was statistically significant (*p* < 0.05), and the observed effect of IgY treatment was dose-dependent.

#### 2.3.3. *Salmonella* spp.

**Figure 4:**
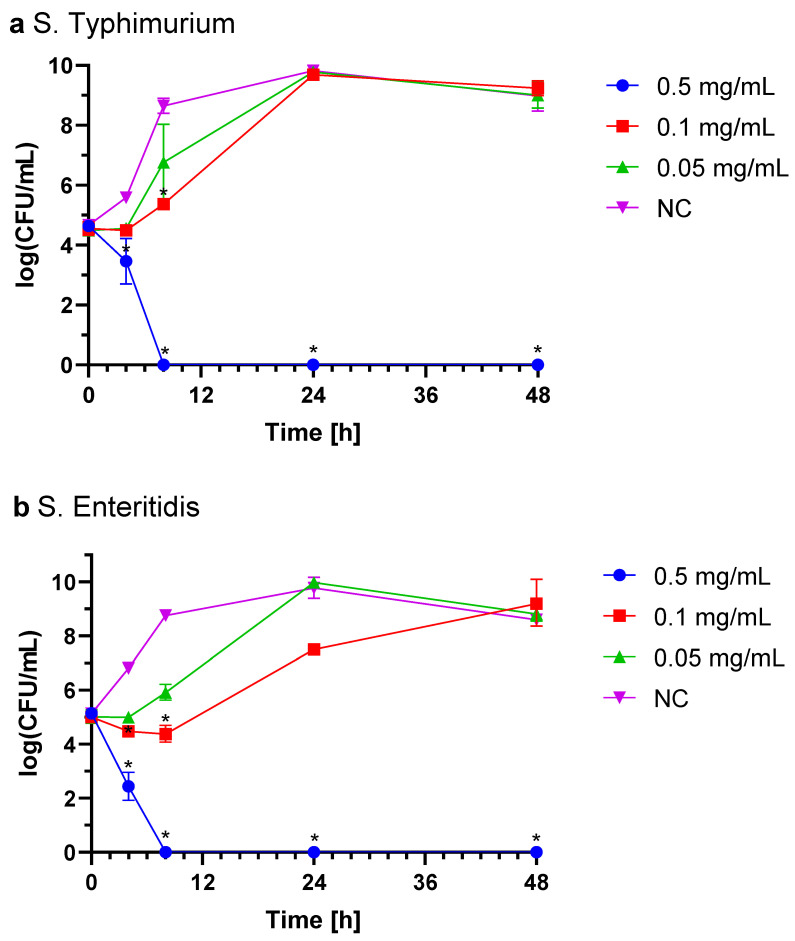
*Salmonella* Typhimurium (**a**) and Enteritidis (**b**) growth with three equal doses of IgY (0.5 mg/mL, 0.1 mg/mL, or 0.05 mg/mL) added at time of inoculation (0 h) or 8 h and 24 h after inoculation. NC, non-treated control; CFU, colony forming unit. Statistically significant differences compared to the NC are marked with * (*p* < 0.05).

The growth inhibition assay for *Salmonella* strains (Typhimurium and Enteritidis) followed a similar dosing regimen. In untreated controls, bacterial counts increased by 4 log CFU, whereas the strongest inhibitory effect on both strains was a 5 log CFU reduction when 0.5 mg/mL IgY concentration was used. At 0.1 mg/mL, partial inhibition was noted for S. Enteritidis after two doses, though growth was not entirely halted (Figure 4a,b). The observed effect was dose-dependent, with lower concentrations yielding reduced inhibitory outcomes. The same IgY concentration was sufficient to inhibit the growth of both *Salmonella* serovars tested. The reduction was statistically significant (*p* < 0.05).

## 3. Discussion

Avian Y immunoglobulins are by far the most widely used for the development of diagnostic tests because they are stable and do not cross-react with important diagnostic factors, such as rheumatoid factor. They also lack binding affinity to complement, Fc receptors, and proteins A or G [10] It is therefore important to point out the possibility of using IgY not only in diagnostics but also as a therapeutic and protective agent in various organisms, which could replace or reduce the overuse of antibiotics [8]. There have been several reports considering the effectiveness of avian Y immunoglobulins for passive immunization against bacterial pathogens. Its therapeutic potential has been confirmed in a variety of in vitro [11,12,13,14] and in vivo studies [12,15,16], establishing its utility in combating specific bacterial infections. Oral administration of avian immunoglobulins as passive immunization may be the most promising IgY application, especially against gastrointestinal tract pathogens [8].

In the current study, we have analyzed the ability of polyclonal IgY antibodies isolated from egg yolk to inhibit the growth of three of the most common foodborne pathogens: *C. jejuni*, Shiga-producing *E. coli* (STEC), and *Salmonella* spp. These three pathogens caused 178,094 incidences of zoonotic disease in humans in 2020 [3]. We successfully isolated and purified specific polyclonal IgY antibodies against all antigens via a modified water dilution method. A key innovation of this study is the use of field-isolated strains of *C. jejuni*, *E. coli*, and *Salmonella* spp., which enhances the clinical relevance of our findings compared to studies relying on laboratory reference strains. This approach ensures the applicability of the results to real-world agricultural scenarios and provides a more accurate assessment of IgY’s efficacy against naturally occurring zoonotic pathogens. The results of our study indicate that polyclonal IgY is effective in reducing the growth and viability of the tested zoonotic pathogens in vitro.

The crucial difference between the pathogens tested in our study was their growth rates. *C. jejuni* is a microaerophilic bacterium with a longer generation time; approximately 2 h is required for bacteria to double in number [17]. The doubling times for *E. coli* and *Salmonella* are about 20 and 30 min, respectively [18,19]. To reduce the effect of this difference, we used an initial CFU of 10^6^ for *C. jejuni* and 10^5^ CFU for *E. coli* and *Salmonella* spp. As a consequence of its longer generation time, *C. jejuni* required only one dose of IgY at 0.5 mg/mL to achieve significant inhibition. Lower IgY concentrations (0.1 mg/mL and 0.05 mg/mL) also demonstrated inhibitory effects, albeit to a lesser extent. Conversely, faster-growing bacteria such as *E. coli* and *Salmonella* spp. necessitated multiple doses at higher IgY concentrations to achieve comparable effects. The dose-dependent efficacy observed in this study aligns with previous findings on IgY applications. For example, Lee et al. [20] and Bustos et al. [12] also observed an inhibitory effect on the growth of *Salmonella* Enteritidis and Typhimurium and *Salmonella* Newport, respectively. Our results demonstrate that IgY exhibits a significant bacteriostatic effect on major zoonotic pathogens, providing an alternative to conventional antibiotics. This aligns with prior studies on IgY’s antimicrobial properties and highlights its potential to mitigate antibiotic resistance in animal husbandry. Notably, the observed efficacy against *C. jejuni* with a single dose suggests practical applicability for slow-growing pathogens, while the dose-dependent effects against *E. coli* and *Salmonella* spp. indicate adaptability for varying bacterial growth dynamics.

By demonstrating IgY’s ability to inhibit growth of zoonotic pathogens, this study addresses two critical global challenges: the overuse of antibiotics in animal husbandry and the increasing prevalence of antimicrobial resistance. Implementing IgY-based interventions in livestock management could contribute to safer food systems and public health.

In conclusion, IgY holds significant promise as a sustainable and ethical alternative to antibiotics in addressing zoonotic diseases, particularly within the One Health framework. Its oral administration as a feed or water additive could offer a practical and non-invasive method for controlling pathogens such as *C. jejuni*, *E. coli*, and *Salmonella* spp. in livestock. Further studies are needed to expand on these findings and explore the potential for large-scale implementation of IgY in agriculture and veterinary medicine. IgY treatment may be especially beneficial against long-lived pathogens such as microaerophilic *C. jejuni*. Moreover, the production and isolation of large quantities of good quality IgY is cost effective, economically advantageous and should be considered as a valuable source of effective and safe antibiotic substitutes. Given its stability, scalability, and ethical production, IgY has the potential to reduce reliance on antibiotics and combat antimicrobial resistance. Despite these promising findings, the study is not without limitations. For instance, the experimental design relied solely on in vitro assays, which, while informative, may not fully replicate the complex interactions occurring in vivo. Future studies will focus on validating these findings in vivo and exploring commercial-scale IgY applications.

## 4. Materials and Methods

### 4.1. Bacteria Strains and Growing Conditions

All bacterial strains used in this study were isolated from field samples collected on poultry and swine farms in Poland and are listed in Table 2. Field-isolated strains were used to ensure the clinical relevance of the findings and their applicability to real-world scenarios in animal husbandry.

#### 4.1.1. *Campylobacter jejuni*

Samples were obtained from diarrheic chickens on Polish poultry farms. Cloacal swabs were placed in Amies medium and transported to the laboratory at 2–8 °C. Not later than 5 h after collection, the swabs were inoculated in BHI medium (BioMaxima, Lublin, Poland) and incubated (48 h, 42 °C, microaerophilic conditions: 85% N_2_, 10% CO_2_, 5% O_2_) in a MCO-170MUV CO_2_/N_2_ incubator (Phcbi, Breda, The Netherlands). Bacteria were then plated on CCDA agar plates (BioMaxima, Poland) and incubated (48 h, 42 °C, microaerophilic conditions). Single colonies were inoculated on Brillance Campy medium (Thermo Scientific, Waltham, MA, USA) and incubated as described above. Suspected colonies were observed by phase-contrast light microscopy to confirm the desired morphology (motile, spiral rod). Typical colonies were confirmed using latex agglutination reagent (Microgen Bioproducts, Eastleigh, UK). Biochemical and genetic characteristics were assessed using the API CAMPY test (bioMerieux, Craponne, France) and PCR, respectively (PCR profiles, primer sequences, and conditions are listed in Supplementary Table S1) [21,22,23,24,25,26,27].

#### 4.1.2. *Escherichia coli*

Samples were obtained from diarrheic piglets on Polish swine farms. Rectal swabs were placed in Amies medium and transported to the laboratory at 2–8 °C. The swabs were inoculated in MacConkey broth (BioMaxima, Poland) and incubated (24 h, 37 °C, 150 rpm). Bacteria were then plated on CHROMagar Coliform selective chromogenic medium (Chromagar, Paris, France) and incubated (24 h, 37 °C). Suspected colonies were identified using slide agglutination with diagnostic sera (Sifin, Berlin, Germany) and PCR for specific genes (e.g., 16S rRNA, stx2e). PCR profiles, primer sequences, and conditions are listed in Supplementary Table S2 [28,29].

#### 4.1.3. *Salmonella* spp.

*Salmonella* spp. isolates were field strains from Immunolab’s private collection.

#### 4.1.4. Storage and Handling

All confirmed strains were stored at −80 °C in storage media (15% glycerol in MHB or TSB, depending on species). Prior to experimental use, strains were revived and cultured under optimal growth conditions specific to each bacterial species.

### 4.2. Antigen Preparation for Hen Immunization

#### 4.2.1. *Campylobacter jejuni*

*C. jejuni* strain CJ6 from glycerol broth was plated on CCDA plates and incubated (48 h, 42 °C, microaerophilic conditions). Material from the plates was collected and suspended in MH broth (BioMaxima, Poland). An Applikon Bio glass bioreactor (Applikon, Schiedam, The Netherlands) filled with MH broth was inoculated with the suspension at a ratio of 1:100 and cultured (24 h, 42 °C, 250 rpm, microaerophilic conditions).

#### 4.2.2. *Escherichia coli*

An *E. coli* K3 strain from glycerol broth was inoculated into LB medium and incubated (24 h, 37 °C, 150 rpm). A glass bioreactor, Applikon Bio (Applikon, The Netherlands), filled with LB broth was inoculated with suspension at a ratio of 1:200 and cultured (24 h, 37 °C, 250 rpm, 0.5 L/min AIR).

#### 4.2.3. *Salmonella* spp.

*Salmonella* Kapemba or Paratyphi B from the master strain was inoculated into *Salmonella* Enrichment Broth (BioMaxima, Poland) and incubated (6 h, 37 °C, 150 rpm). The suspension was then plated, spread evenly on agar plates, and incubated (24 h, 37 °C). The agar surface was washed with sterile 0.85% NaCl to collect bacteria and the suspension was transferred to a sterile flask.

#### 4.2.4. Bacteria Inactivation

Bacteria of all strains were inactivated with the use of 0.5% buffered formaldehyde (pH 8.0). Suspensions were transferred to flasks, formaldehyde was added, and the flasks were sealed with rubber stoppers before being incubated (24 h, 37 °C, 150 rpm). Bacteria were harvested by centrifugation (20 min, 5000× *g*) and resuspended in sterile 0.85% NaCl with 0.02% buffered formaldehyde (pH 8.0). Inactivation was confirmed by inoculating the suspension into *Salmonella* Enrichment Broth (Biomaxima, Poland) and tryptic soy broth and incubating (14 days, 37 °C). Bacterial counts were determined with the use of turbidity measurement using a Den-1b densitometer (Biosan, Riga, Latvia). Conversion from turbidity (McFarland standard) to CFU was performed using an experimentally determined growth curve for each bacterial species.

### 4.3. Hen Immunization Protocol

A total of 100 Leghorn hens (4–6 weeks old) were divided into four groups (*n* = 25 per group) and immunized with inactivated bacterial antigens: CJ6, K3, SK, or SP (Table 1). Immunization via intramuscular injection was conducted at two-week intervals. Four 1 mL doses were administrated: the first dose contained 3 × 10^9^ CFU with Freund Complete Adjuvant, while subsequent doses contained 6 × 10^9^ CFU with Freund Incomplete Adjuvant. No control group was included in order to minimize the number of animals used in the experiment, and IgY levels were compared to those in commercial eggs. Eggs were collected daily for two weeks following the final dose and stored at 4 °C until antibody isolation. The condition of all hens was monitored throughout the experiment and at the end of egg collection. Hens that were not euthanized at the end of the experiment were transferred to a local farm for further breeding. The experiments were approved by the Local Ethical Committee for Animal Experiments in Olsztyn under Resolution No. 38/2021, dated 19 May 2021. All procedures complied with the relevant ethical guidelines and regulations. The study adheres to the ARRIVE guidelines for reporting animal research.

### 4.4. IgY Isolation and Purification

The water-soluble fraction (WSF) containing IgY was isolated from the egg yolk using a modified water dilution method [30,31]. Briefly, egg yolks were diluted (1:10) with distilled water, adjusted to a pH of 5.0, and stored overnight at 4 °C. After centrifugation (11,000× *g*, 30 min, 4 °C), the WSF was collected. The WSF was mixed with 0.01% activated carbon (Chempur, Piekary Slaskie, Poland), incubated for 30 min (RT), centrifuged (11,000× *g*, 30 min, 4 °C), and filtered through filter paper (Whatman, Kent, UK). WSF was concentrated using an automatic ultrafiltration system (Sartorius, Goettingen, Germany) and a dedicated 100 kDa pore size cassette. Then, 1.5 M NaCl was added for precipitation and incubated (2 h, RT) while mixing. The precipitate was centrifuged (14,000× *g*, 20 min, 4 °C) and resuspended in saline, filtered through 0.22 µm pore Puradisc PES filters (Whatman, UK), and stored at 4 °C. IgY concentrations were determined by measuring the absorbance at 280 nm with a Multiscan SkyHigh spectrophotometer (Thermo Scientific, USA), using an extinction coefficient of 1.33 mL/mg for IgY.

### 4.5. Purity Assessment (SDS-Page)

Purified antibodies were analyzed using 12% sodium dodecyl sulfate polyacrylamide gel electrophoresis (SDS-PAGE) under reducing conditions. The electrophoresis was conducted in two steps: 100 V for 20 min, followed by 160 V for 60 min. Prior to electrophoresis, the samples were diluted in Laemmli sample buffer (Sigma-Aldrich, St Louis, MO, USA) and heated in a Thermomixer C (Eppendorf, Hamburg, Germany) at 100 °C for 5 min. Twenty-five microliters of each sample were loaded into the wells, alongside a pre-concentrated protein marker (Protein Ladder EXtended 5–245 kDa, Geneon, Ludwigshafen, Germany) as the molecular weight standard. A chromatography-purified normal chicken IgY standard (20 mg/mL; Gallus Immunotech, Wildwood, MI, USA) was used as a positive control.

### 4.6. IgY Titer Assessment (Tube Agglutination Test)

An agglutination test to detect specific IgY antibodies against *C. jejuni*, *E. coli*, and *Salmonella* O:B and O:D antigens was performed in round-bottomed tubes. Briefly, twofold serial dilutions of IgY isolates (1:10 to 1:1280) were mixed with inactivated antigen at a final concentration of two McFarland units (measured with a Den-1b densitometer, Biosan, Latvia). The mixtures were vortexed and incubated overnight at 37 °C. The titer was defined as the highest dilution at which agglutination was observed.

### 4.7. Growth Inhibition Assay

#### 4.7.1. *Campylobacter jejuni*

Bacteria were inoculated onto CCDA plates and incubated (48 h, 42 °C, microaerophilic conditions). Bacteria were harvested from the agar surface and suspended in MH broth. The suspension was inoculated into flasks to achieve a concentration of 10^6^ CFU/mL. One dose of IgY was added to reach final concentrations of 0.5 mg/mL, 0.1 mg/mL, or 0.05 mg/mL at 0, 8, or 24 h post-inoculation. Flasks were incubated on a shaker (48 h, 42 °C, microaerophilic conditions). Bacterial cell counts were determined by plating serial dilutions on CCDA plates using the EasySpiral Pro automatic plater (Interscience, Saint-Nom-la-Breteche, France), with three replicates for each dilution. Plates were incubated under the same conditions (42 °C, 48 h), and colonies were counted using the Scan500 automated counter (Interscience, France).

#### 4.7.2. *Escherichia coli* and *Salmonella* spp.

Bacteria were cultured in TSB medium and incubated (24 h, 37 °C, 150 rpm). The suspension was transferred into flasks to achieve a concentration of 10^5^ CFU/mL, which was lower than for *C. jejuni* due to the higher growth rates of *E. coli* and *Salmonella* spp. Three doses of IgY were added to reach final concentrations of 0.5 mg/mL, 0.1 mg/mL, or 0.05 mg/mL at 0, 8, and 24 h post-inoculation. Sterile 0.85% NaCl was added to the untreated control. Flasks were incubated (48 h, 37 °C, 150 rpm). Bacterial cell counts were determined by plating serial dilutions on agar plates (LA for *E. coli* and TSA for *Salmonella Enteritidis* and *Typhimurium*) using the EasySpiral Pro automatic plater (Interscience, France), with three replicates per dilution. Plates were incubated at 37 °C for 24 h, and colonies were enumerated using the Scan500 automated counter (Interscience, France).

#### 4.7.3. Statistical Analysis

Statistical analysis was carried out using data analysis software STATISTICA version 13.3 (StatSoft, Inc., Tulsa, OK, USA). All quantitative data are presented as median values with minimal to maximal range. The analysis of variance was employed to determine whether there were statistically significant differences in the inhibitory effects of IgY on the growth of the tested pathogens. This method facilitated comparisons between multiple groups, specifically assessing the impact of different IgY concentrations on bacterial viability. Following ANOVA, post-hoc tests, such as Tukey’s test, were utilized to identify specific group differences. This step was crucial for understanding which concentrations of IgY were most effective at inhibiting bacterial growth.

## Figures and Tables

**Table 1:** Specific IgY titers in IgY egg yolk isolates.

IgY Type	Antigen Used for Agglutination	Titer (Highest Dilution with Visible Agglutination)
IgY-CJ	CJ6	1:320
IgY-OB	S. Typhimurium	1:640
IgY-OD	S. Enteritidis	1:640
IgY-EC	K3	1:320

**Table 2:** Bacteria strains used in experiments.

Symbol	Species	Description
CJ6	*Campylobacter jejuni*	CDT, CadF, CiaB, FlgR
K3	*Escherichia coli*	O:139, F5, Stx2e, STb
ST	*Salmonella* serovar Typhimurium	[1, 4, 12 : i : 1, 2] O:B
SE	*Salmonella* serovar Enteritidis	[1, 9, 12 : gm ; -] O:D
SK	*Salmonella* serovar Kapemba	[1, 9, 12 : lv : 1, 7] O:D
SP	*Salmonella* serovar Paratyphi B	[1, 4, 12 : b : 1, 2] O:B

## Data Availability

The authors confirm that all data supporting the findings of this study are included within the article. Additional data are available upon reasonable request from the corresponding author, K.B. However, the data are not publicly accessible due to company confidentiality restrictions.

## Supplementary Materials

The following supporting information can be downloaded at: https://www.mdpi.com/article/10.3390/ijms26031040/s1.
