# Supplementary material for: Growth-Inhibitory Effect of Chicken Egg Yolk Polyclonal Antibodies (IgY) on Zoonotic Pathogens Campylobacter jejuni, Salmonella spp. and Escherichia coli, In Vitro
Source: Int J Mol Sci. 2025 Jan 25;26(3):1040. doi: 10.3390/ijms26031040 (PMC11816624; doi:10.3390/ijms26031040)
Supplement: Supplementary file 1 [file ijms-26-01040-s001.zip › Supplementary Tables S1 and S2.pdf]

Table S1. PCR profiles used for C. jejuni identification - DNA was isolated using the Genomic Mini AX Bacteria Kit (A&A Biotechnology, Gdynia, Poland). PCR Mix Plus Green 2x (A&A Biotechnology, Poland) containing Taq polymerase (0.1 U/µl), MgCl2 (4 mM), dNTPs (0.5 mM each), blue and yellow dye and loading buffer was mixed with 0.4 µM forward and reverse primer, 0.1 µg of DNA for each reaction and filled with sterile PCR water. PCR was performed on a Labcycler thermal cycler (Sensoquest, Goettingen, Germany).

Gene	Primer sequence 5′-3′ (F – forward, R – reverse)	Amplicon size	Cycles number	Denaturation	Annealing	Elongation	Ref
16S rRNA	F: ATCTAATGGCTTAACCATTAAAC<br>R: GGACGGTAACTAGTTTAGTATT	857 bp	35	30s, 95°C	1m30s, 59°C	1m, 72°C	[21]
flaA	F: GGATTTCGTATTAACACAAATGGTGC<br>R: CTGTAGTAATCTTAAAACATTTTG	1728 bp	35	30s, 92°C	1m30s	2m30s, 72°C	[22]
flaA/flaB	F: CTTCAGGGATGGCGATAGCAGATA<br>R: TTGATCTCTTCAGCCAAAGCTCCAAGT	533 bp	40	1m, 94°C	1m, 60°C	2m, 72°C	[23]
flaC	F: TAATACGACTCACTATAGGGAT GATGATCTCTGATGCAACTATGA<br>R: AAAGCAGCAGCATTTTCTTTTAGAT	712 bp	35	30s, 95°C	1m, 50°C	1m, 72°C	[24]
cdtA	F: CCTTGTGATGCAAGCAATC<br>R: ACACTCCATTTGCTTTCTG	370 bp	30	1m, 94°C	1m, 49°C	1m, 72°C	[25]
cdtB	F: CAGAAAGCAAATGGAGTGTT<br>R: AGCTAAAAGCGGTGGAGTAT	620 bp	30	1m, 94°C	1m, 51°C	1m, 72°C	[25]
cdtC	F: CGATGAGTTAAAACAAAAAGATA<br>R: TTGGCATTATAGAAAATACAGTT	182 bp	30	1m, 94°C	1m, 47°C	1m, 72°C	[25]
cadF	F: TTGAAGGTAATTTAGATATG<br>R: CTAATACCTAAAGTTGAAAC	400 bp	35	30s, 95°C	1m, 45°C	1m, 72°C	[25]
ciaB	F: TTTCCAAATTTAGATGATGC<br>R: GTTCTTTAAATTTTTCATAATGC	1165 bp	35	30s, 95°C	1m, 45°C	1m, 72°C	[26]
flgR	F: GAGCGTTTAGAATGGGTGTG<br>R: GCCAGGAATTGATGGCATAG	390 bp	35	30s, 94°C	30s, 60°C	30s, 68°C	[27]

Table S2. PCR profiles used for E. coli STEC identification - DNA was isolated from suspected colonies using the Genomic Mini AX Bacteria Kit (A&A Biotechnology, Poland). PCR Mix Plus Green 2x (A&A Biotechnology, Poland) containing Taq polymerase (0.1 U/µl), $MgCl_2$ (4 mM), dNTPs (0.5 mM each), blue and yellow dye and loading buffer was mixed with 0.4 µM forward and reverse primer, 0.1 µg of DNA and filled with PCR water for each reaction. PCR assays were performed on a Labcycler thermal cycler (Sensoquest, Germany) to confirm species and immunogenic characteristics.

Gene/ product	Primer sequence 5′-3′ (F – forward, R – reverse)	Amplicon size	Cycles number	Denaturation	Annealing	Elongation	Ref
16S rRNA	F: GGAAGAAGCTTGCTTCTTTGCTGAC<br>R: AGCCCGGGGATTTCACATCTGACTTA	544 bp	40	45 s, 94°C	45 s, 72°C	45 s, 72°C	[28]
stx2e	F: ATGAAGAAGATGTTTATAGCGGT<br>R: GTTAAACTTCACCTGGGCAAA	264 bp	35	30 s, 95°C	30 s, 42°C	15 s, 72°C	This work
fanA	F: CACTTGAGGGTATATGCGATCTT<br>R: GACCTCAGTCACAGCAACTATAC	92 bp	45	10 s, 95°C	30 s, 62°C	25 s, 72°C	[29]
STa	F: ATGAAAAAGCTAATGTTGGC<br>R: TACAACAAAGTTCACAGCAG	193 bp	45	10 s, 95°C	30 s, 60°C	25 s, 72°C	[29]
STb	F: TGCCTATGCATCTACACAAT<br>R: CTCCAGCAGTACCATCTCTA	113 bp	45	10 s, 95°C	30 s, 60°C	25 s, 72°C	[29]
