# Supplementary figures and images for: Growth-Inhibitory Effect of Chicken Egg Yolk Polyclonal Antibodies (IgY) on Zoonotic Pathogens Campylobacter jejuni, Salmonella spp. and Escherichia coli, In Vitro
Source: Int J Mol Sci. 2025 Jan 25;26(3):1040. doi: 10.3390/ijms26031040 (PMC11816624; doi:10.3390/ijms26031040)

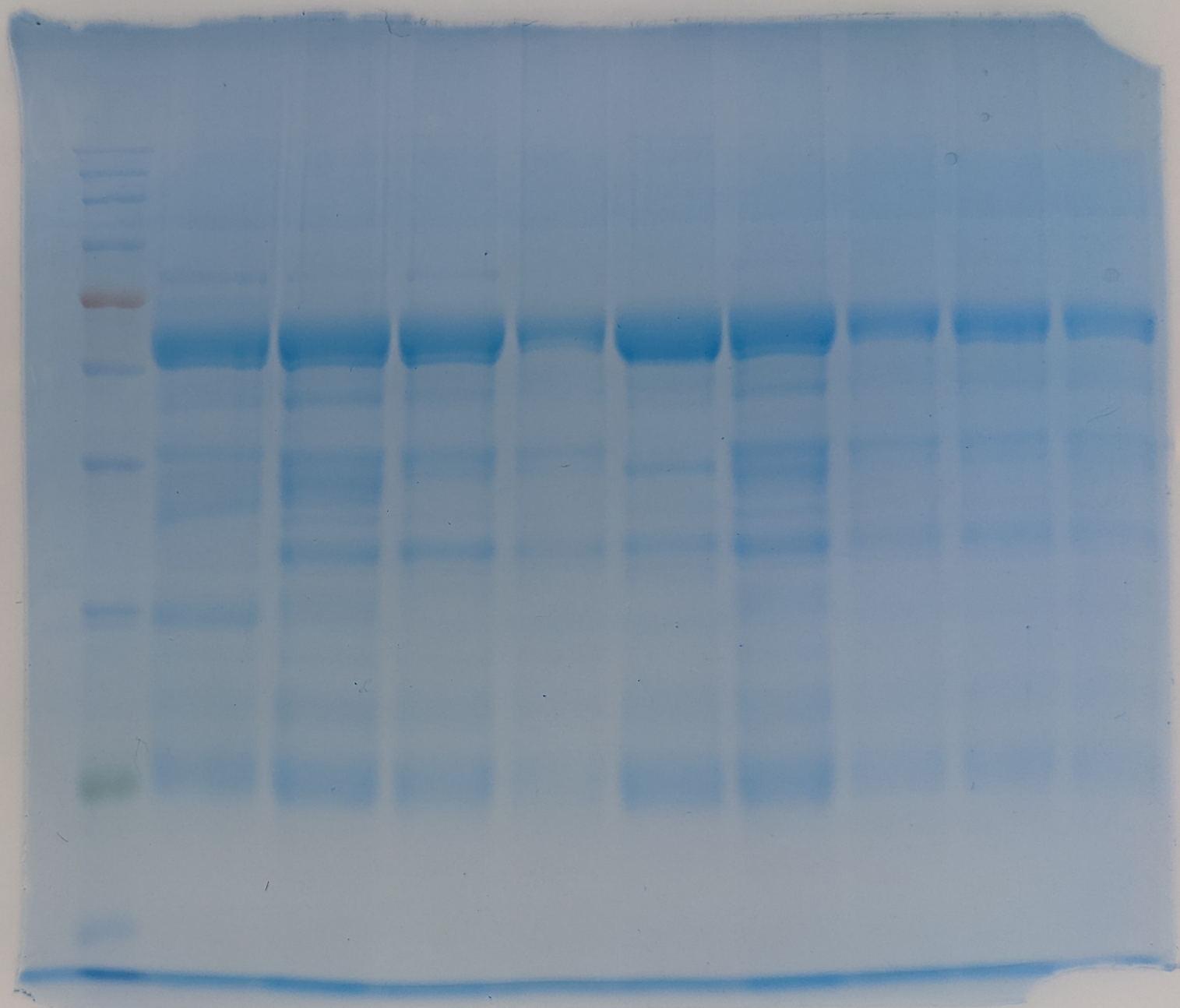

Supplement: Supplementary file 1 [file ijms-26-01040-s001.zip › Supplementary Figure S1.pdf]
